# In Situ Monitored (N, O)‐Doping of Flexible Vertical Graphene Films with High‐Flux Plasma Enhanced Chemical Vapor Deposition for Remarkable Metal‐Free Redox Catalysis Essential to Alkaline Zinc–Air Batteries

**DOI:** 10.1002/advs.202200614

**Published:** 2022-03-04

**Authors:** Zhiheng Wu, Yuran Yu, Gongkai Zhang, Yongshang Zhang, Ruxin Guo, Lu Li, Yige Zhao, Zhuo Wang, Yonglong Shen, Guosheng Shao

**Affiliations:** ^1^ State Center for International Cooperation on Designer Low‐Carbon & Environmental Materials (CDLCEM) School of Materials Science and Engineering Zhengzhou University 100 Kexue Avenue Zhengzhou 450001 China; ^2^ Zhengzhou Materials Genome Institute (ZMGI) Building 2, Zhongyuanzhigu, Xingyang Zhengzhou 450100 China

**Keywords:** high‐flux plasma enhanced chemical vapor deposition (HPECVD), in situ codoping, metal‐free ORR and OER catalysis, plasma diagnostics, vertical graphene crystal, zinc–air batteries

## Abstract

Rechargeable zinc–air batteries (ZABs) have attracted great interests for emerging energy applications. Nevertheless, one of the major bottlenecks lies in the fabrication of bifunctional catalysts with high electrochemical activity, high stability, low cost, and free of precious and rare metals. Herein, a high‐performance metal‐free bifunctional catalyst is synthesized in a single step by regulating radicals within the recently invented high‐flux plasma enhanced chemical vapor deposition (HPECVD) system equipped with in situ plasma diagnostics. Thus‐derived (N, O)‐doped vertical few‐layer graphene film (VGNO) is of high areal population with perfect vertical orientation, tunable catalytic states, and configurations, thus enabling significantly enhanced electrochemical kinetic processes of oxygen reduction reaction (ORR) and oxygen evolution reaction (OER) with reference to milestone achievements to date. Application of such VGNO to aqueous ZABs (A‐ZABs) and flexible solid‐state ZABs (S‐ZABs) exhibited high discharge power density and excellent cycling stability, which remarkably outperformed ZABs using benchmarked precious‐metal based catalysts. The current work provides a solid basis toward developing low‐cost, resource‐sustainable, and eco‐friendly ZABs without using any metals for outstanding OER and ORR catalysis.

## Graphical abstract

Highly active metal‐free bifunctional ORR‐OER catalyst fabricated by high‐flux plasma enhanced chemical vapor deposition with in situ plasma diagnostics, thus enabling high‐performance Zn‐air batteries outperforming those using catalysts based on rare and precious metals.

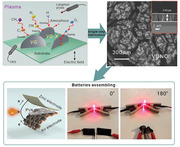

## 1 Introduction

With the rapid increase of energy demands and ever‐pressing environmental crisis, enormous efforts have been made to explore advanced devices for clean energy conversion and storage.^[^ ^1^, ^2^, ^3^, ^4^ ^]^ Rechargeable zinc‒air batteries (ZABs) have attracted tremendous attention owing to the abundant zinc reserve, high theoretical energy density (1086 Wh kg^−1^), and intrinsic safety.^[^ ^5^, ^6^ ^]^ However, their applications are still hindered from inadequate achievement in both power density and energy efficiency, which is largely attributed to inherently sluggish kinetic processes in oxygen reduction reaction (ORR) and oxygen evolution reaction (OER) during discharge and charge processes at the cathode.^[^ ^7^, ^8^ ^]^ In rechargeable ZABs, precious metal‐based catalysts such as Pt and IrO_2_/RuO_2_ are commonly used in the air cathode to accelerate ORR and OER, respectively. However, it has been recognized that the overall performance of ZABs based on such expensive and lean‐resourced catalysts is restricted by their poor stability and complexity in needing to combine ORR and OER catalysts to deliver the bifunctional catalytic activities.

Recently, strenuous efforts have been made in search of alternatives to substitute precious metal based electrocatalysts, among which metal‐free graphene‐like catalysts stand out as highly promising candidates for catalyzing both ORR and OER, since in addition to the almost limitless carbon resource, graphene‐like materials have exhibited great potential in offering both high electrocatalytic activity and robust stability.^[^ ^9^, ^10^ ^]^ Single elemental doping,^[^ ^11^, ^12^ ^]^ dual doping,^[^ ^13^ ^]^ even tri‐doping^[^ ^14^ ^]^ have been adopted to enhance activities and densities of catalytic sites. However, it has been noted that materials based on such graphene‐like powders or chemically reduced graphene oxide are prone to agglomeration with in‐plane stacking, which is vertical to the charge transfer direction for both ORR and OER, thus not desirable for both ion and charge transportations essential to metal‐air batteries operating at static state without forced transport of reactants. Also, their recognized poor crystallinity tends to hinder the full potential of crystalline graphene, in terms of tunable doped states and high conductivities to ions and electrons.

Consequently, vertically aligned graphene arrays (VGs) have been exploited as matrix to anchor active catalytic species and even as alternatives to metal‐based catalysts, due to their unique morphology as well as excellent charge transfer capability.^[^ ^15^, ^16^, ^17^ ^]^ The current‐state‐of‐art strategies for preparing vertical graphene materials involve thermal chemical vapor deposition (TCVD) and plasma enhanced CVD (PECVD).^[^ ^18^, ^19^ ^]^ Benefiting from the low‐temperature and catalyst‐free feature, PECVD has been proved to be a feasible method for VG fabrication with controllable morphology and structure. Nevertheless, typical direct current PECVD (dc‐PECVD)^[^ ^20^ ^]^ suffers from low density of ions in plasma flux, and conventional tube‐type radio frequency PECVD (RF‐PECVD)^[^ ^19^, ^21^ ^]^ has rather poor large area uniformity due to significant decay of plasma intensity over propagation.

Recently, we have invented a high‐flux plasma enhanced chemical vapor deposition (HPECVD) method,^[^ ^22^ ^]^ which relies on effective enhancement of plasma stability with streamlined electromagnetic field beyond extended spatial regions. Such a HPECVD process shows great advantages in a) providing stable plasma with very high ion densities over extended region essential for uniform growth of vertically aligned graphene arrays at low‐temperature without the need of substrate seeding,^[^ ^23^ ^]^ b) permitting versatile controlling parameters such as plasma launching power, magnetic field, in situ doping gases, and working vacuum, which permits tuning of radical species, their densities and kinetic energies, being essential in controlling the nucleation and growth of graphene crystals and even more importantly doped states/configurations,^[^ ^24^, ^25^ ^]^ c) being inherently clean and eco‐friendly to environment, and d) allowing growth of tunable population of VG crystals, helpful to provide well‐exposed catalytic sites in addition to providing effective pathways via aligned transportation of electrons and ions along the basal planes of graphene petals/sheets.^[^ ^26^, ^27^ ^]^ In situ N‐doping of such vertical graphene (VGN) using the HPECVD process demonstrated great potential in offering tunable doping states and VG morphologies and populations, with the synergistic benefit highly effective in delivering enhanced redox processes for ORR.^[^ ^28^ ^]^ It is apparently helpful that the process can be monitored in situ, so that the populations and states of depositing species can be readily tuned towards delivering doped VG films for optimized catalytic performance.

Herein, we have established in‐situ plasma diagnostics to monitor the HPECVD process, which enabled single‐step fabrication of N and O codoped vertical graphene (VGNO) films with advantageous morphologies and preferable doping configurations. Through real‐time plasma monitoring data, the mechanisms and structural/bonding characteristics can be elaborated in terms of constituents, densities, and states of various radical species. The optimized deposition condition helped to co‐dope O and N in the most stable structural configurations consistent with theoretical predictions using the density functional theory (DFT) method, and this in turn resulted in remarkably enhanced ORR and OER catalysis for highly enhanced redox kinetics over VGN, owing to effectively reduced kinetic barriers in alkaline Zn‐air battery cells. Consequently, utilization of the VGNO cathode in both aqueous ZABs (A‐ZABs) and solid‐state ZABs (S‐ZABs) exhibited amazing performance with reference to landmark achievements to date. The VGNO based A‐ZABs exhibited a high peak power density of 221.9 mW cm^−2^ with excellent cycling stability over 300 h at a rather high charge‐discharge current densities over 10 mA cm^−2^, which either evidently outperformed the best reported data at the same level of cathode matter loading, mainly owing to 10% improvement of the open‐circuit voltage; or rivaled the highest reported power density with only half of cathode matter. Also, highly bendable/flexible solid battery cells have been realized using the current VGNO as cathode, delivering encouraging preliminary peak power density over 52.9 mW cm^−2^ within a discharge/charge voltage gap of 0.83 V at 2 mA cm^−2^.

## 2 Results and Discussion

**Figure 1:**
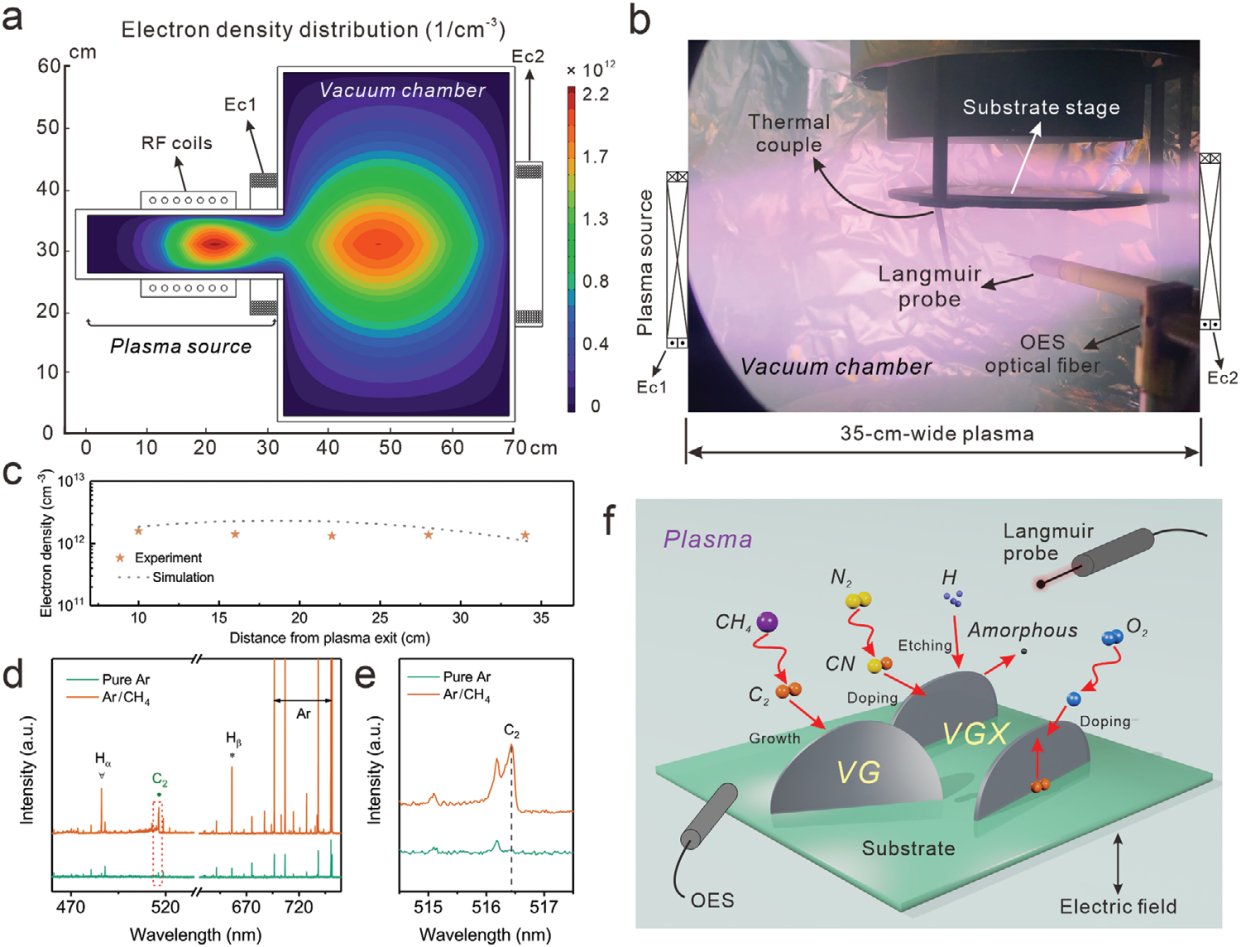
a) Simulated electron density distribution in the HPECVD system consisting of a plasma source, a vacuum chamber, RF coils and two electromagnetic coils (Ec1 and Ec2) in argon plasma at a RF power of 1500 W. b) Photo of the plasma optical emissions in HPECVD. c) Simulated electron density along the axis of the model and experimental results of electron density at a RF power of 1500 W along the axis of the plasma optical emissions in b). d) Optical emission spectra of the Ar and Ar/CH_4_ plasma. e) Comparison of the C_2_ radicals according to the selected region in d). f) Schematic illustration for the growth of vertically standing graphene (VG) and heteroatom (X) doped vertically standing graphene (VGX) from reactive radicals.

Finite element method was employed to simulate the magnet field and electron density distribution in HPECVD system. A 2D model was constructed as shown in **Figure** **1**a, consisting of a plasma source, a vacuum chamber, RF coils and two electromagnetic coils (Ec1 and Ec2). The scale of the model is proportional to the processing equipment. Electromagnet field was to be streamlined with a magnetic mirror configuration to guide and confine plasma for long‐range propagation (Figure S1, Supporting Information). Based on such a geometric design, a static magnetic field was introduced in the inductively coupled plasma (ICP), further enhancing the electron cyclotron resonance (ECR) absorption.^[^ ^29^ ^]^ The electron density was simulated as a key parameter to dictate the plasma density. As shown in the simulated result, high plasma density was obtained both at the plasma source and the plasma chamber in the HPECVD system. As is shown in Figure 1b, the high‐density plasma in our HPECVD system propagates along the aligned axis of magnetic coils beyond the substrate stage. In contrast, the inductively coupled plasma in a normal PECVD without extra electromagnetic field shows gradually decaying electron density and the electron density in the vacuum chamber is nearly two orders of magnitude lower than that in our HPECVD system (Figure S2, Supporting Information). The high electron density is critical for effective ionization, excitation, and dissociation of the introduced gases,^[^ ^22^, ^23^, ^30^, ^31^, ^32^ ^]^ endowing high densities of reactive ions and radicals with adequate kinetic energy to promote in situ crystallization.

Under the guidance of simulation, we have established an in situ plasma diagnostic system containing a Langmuir probe and optical emission spectrometer (OES) to measure the electron density and identify radicals from the plasma flux, which enables monitoring and tuning of plasma condition via varying processing parameters (Figure S3, Supporting Information). Figure 1c shows the electron density measured along the axis of the plasma flux (shown in Figure 1b), with comparison with the simulated result along the axis of the model. It is apparent that the electron density is up to 1.6 × 10^12^ cm^−3^, which is about three orders higher than that of dc‐induced plasma and over ten times of that in RF‐plasma.^[^ ^29^, ^33^ ^]^ Meanwhile, the electron density distribution is quite uniform over the 25 cm propagation pathway between the aligned two magnetic coils, which is consistent with the simulation results. These beneficial features of the HPECVD plasma contribute to the substrate‐independent nucleation and growth of vertical graphene at rather low substrate temperature without heater (Figure S4, Supporting Information).^[^ ^23^ ^]^

Figure 1d shows the typical optical emission spectra of Ar/CH_4_ and Ar plasma obtained by the OES. The spectra contain various emission lines, including C_2_ (516.4 nm), H_*α*_ (656 nm), H_*β*_ (486 nm), and Ar lines.^[^ ^34^ ^]^ As shown in Figure 1e, the C_2_ lines in a typical Ar/CH_4_ plasma is well defined, which is absent in the Ar plasma, indicating abundant C_2_ radicals being generated in our HPECVD system. This is of vital importance for the nucleation and growth of graphene sheets/petals.^[^ ^35^, ^36^ ^]^ The tailoring of the contents and densities of the C_2_ radicals with respect to other activated species in the plasma is essential in determining the formation of VG structures, as is illustrated in Figure 1f.

**Figure 2:**
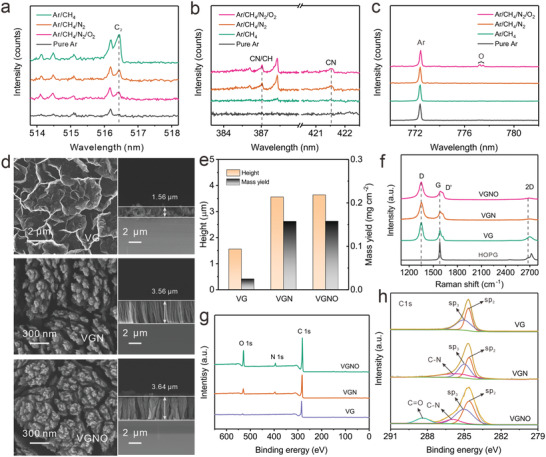
a–c) Typical optical emission spectra (OES) of Ar, Ar/CH_4_, Ar/CH_4_/N_2_, and Ar/CH_4_/N_2_/O_2_ plasma at different wavelength. d) SEM images of as fabricated samples in Ar, Ar/CH_4_, Ar/CH_4_/N_2_, and Ar/CH_4_/N_2_/O_2_ plasma (VG, VGN, and VGNO, respectively). e) Histogram comparing the height and mass yield of VG, VGN, and VGNO in d). f) Raman spectra of VG, VGN, VGNO, and highly oriented pyrolytic graphite (HOPG) for reference. g) XPS survey spectra and h) High‐resolution XPS spectra of C1s for VG, VGN, and VGNO.

OES is a powerful tool for in situ monitoring and quantification of radicals in plasma during material formation.^[^ ^37^ ^]^ **Figure** **2**a–c shows optical emission spectra in Ar, Ar/CH_4_, Ar/CH_4_/N_2_, and Ar/CH_4_/N_2_/O_2_ plasma fluxes. It is shown that the intensity of C_2_, CN, CN/CH, and O can be tailored simply by varying introduced gases. The Ar/CH_4_ plasma shows the highest intensity of C_2_ peak, which is less strong in the Ar/CH_4_/N_2_/O_2_ plasma and neglectable in the Ar plasma. The intensities of CH/CN and CN radicals increase with the introduction of feedstock gas, and the O radicals is present in Ar/CH_4_/N_2_/O_2_ plasma due to the introduction of oxygen gas. Meanwhile, the individual OES spectrum in different plasma during the growth process shows neglectable difference, indicating stable composition of radicals in a stable plasma environment (Figure S5, Supporting Information). The OES intensity of each radical species is proportional to its content and it is the C_2_ radicals that contribute to forming the sp^2^ C—C bonding as fundamental units in graphene structures, so that the reduced C_2_ radical content due to increase of CN and O radicals would contribute to inhibit the formation of *π* bonding and *π* ^*^ anti‐bonding orbitals, leading to reduced sp^2^ bonded cluster/domain sizes.^[^ ^38^, ^39^ ^]^ SEM images of VG, VGN, and VGNO fabricated in Ar/CH_4_, Ar/CH_4_/N_2_, and Ar/CH_4_/N_2_/O_2_ plasma are shown in Figure 2d. The deposited graphene film was made of a low population of largely inclinedly oriented graphene sheets, with an overall film thickness (i.e., the vertical height) of 1.56 µm after 2 h growth for VG film. In contrast, with the introduction of nitrogen and oxygen in plasma, the morphology evolved into well aligned structure with significantly higher populations of vertical graphene sheets, thus resulting in more than doubled film thickness, 3.56 µm for VGN and to 3.64 µm for VGNO. More importantly, the latter films with well‐aligned vertical graphene sheets are advantageous for redox catalysis of interest to metal air batteries and fuel cells, since the well‐aligned graphene sheets can provide highways for both ionic transportation and transfer of electric charges at the controlling electrode‐electrolyte interfaces to lower the activation energy for the kinetic processes.^[^ ^40^ ^]^ The morphology differences between VG and VGN, VGNO were also confirmed by TEM images (Figure S6, Supporting Information), with the few‐layer graphene structures for VGN and VGNO being confirmed by HRTEM.

The film thickness (or the vertical height of the graphene sheets) was also closely correlated to the overall mass yield. As shown in Figure 2e, pristine VG had a rather low mass yield of 0.025 mg cm^−2^. In comparison, VGN and VGNO had much higher mass yield values of 0.1575 and 0.1586 mg cm^−2^, respectively, owing to their much higher nucleation dictated areal populations, which resulted in preferred vertical orientation advantageous for the advancement of the front edges, leading to faster enlargement of the basal planes of graphene sheets and enhanced utilization of feeding radicals in plasma. One notes that the introduction of doping species into the plasma lead to lowered content of the C_2_ radicals, which together with other species such as CN, and O radicals determines the interplay between nucleation and growth of graphene sheets. While high content of C_2_ radicals promotes both nucleation and growth of graphene, the latter was dominant when there was higher feeding of growth materials so that the substrate would soon be shaded by fast growth of sheets from the initial inclinedly oriented nuclei. The nucleation of graphene at rather low temperatures in a vapor deposition process is typically time‐dependent due to limited diffusion at low substrate temperature.^[^ ^32^ ^]^ Consequently slower growth would allow more time for higher areal nucleation population, such that only those in the well‐defined vertical orientation had sustained opportunity to extend the exposed front edges. Consistently, the CN and O radicals tended to promote sp^3^ bonding owing to moderate effect in bonding energetics, which would contribute to somewhat slowed growth. Overall, high density of vertically orientated graphene sheets led to aligned advancement of their front edges, and resulted in effectively doped, hence more defective and thinner few‐layer graphene sheets, together with enhanced consumption of reactant radicals. In contrast, the inclined graphene sheets in the case of pristine VG offered outer surface to receive condensing C_2_ radicals, thus leading to thicker and bigger graphene sheets (Figure S6, Supporting Information).

The effect of N and O codoping can be readily investigated by comparing Raman spectra, and as is shown in Figure 2f, the spectra from VG, VGN, and VGNO show characteristic Raman peaks of graphene at 1349 cm^−1^ (D peak) due to defects, 1582 cm^−1^ (G peak) from sp^2^ bonding, and 2690 cm^−1^ (2D peak) owing to crystallinity. The intensity ratio of D peak to G peak (*I*_D_/*I*_G_) is a well‐accepted indicator of defect and/or doping levels due to their effects on lattice distortion and the extent/range of sp^2^ bonding. The *I*_D_/*I*_G_ increases from 1.76 for pristine VG to 2.01 for VGN, and 2.12 for VGNO. The more evident D’ peak for doped graphene is also consistent with weakened 2D peak, which is attributed to doping induced imperfection in the graphene lattices.^[^ ^41^ ^]^ As is demonstrated in the HREM results (Figure S6, Supporting Information), the long‐range crystalline lattices of graphene are maintained, confirming again that the double resonant Raman peak 2D is much more sensitive to impurity or imperfection in the crystal lattice of graphene.

The chemical configurations in VG, VGN, and VGNO can be compared with X‐ray photoelectron spectroscopy (XPS). The XPS survey spectra clearly show C, O, and N peaks for VGNO, with high atomic content of 16.7% for oxygen and 5.1% for nitrogen (Figure 2g). In contrast, the O peak is quite weak in VGN and almost absent in the VG sample. The high‐resolution C 1s spectra (Figure 2h) can be deconvoluted into peaks located at 284.6, 285.0, 286.0, and 288.4 eV, which are attributed to C—C sp^2^, C—N, C—C sp^3^, and C═O bonding, correspondingly. Apparently, the C═O bonding is present in VGNO and absent in VG and VGN, while, the C—N bonding exists in VGN, VGNO but not in VG, being consistent with the radicals present in the corresponding plasma fluxes. The high‐resolution N 1s spectra exhibit three different N doping configurations, pyridinic N (398.3 eV), pyrrolic N (400.1 eV), and graphitic N (402.7 eV) (Figure S7, Supporting Information) in VGN and VGNO,^[^ ^26^ ^]^ with the pyridinic N being the major constituent.

**Figure 3:**
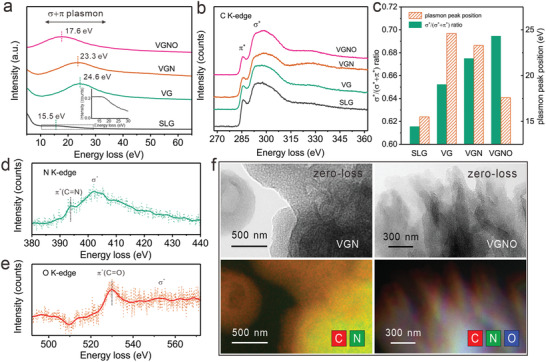
Energy loss C spectra in VG, VGN, VGNO, in comparison to that of perfect single layer graphene (SLG): a) low energy plasmon spectra, and b) C K‐edge at high energy range. The inset in a) is the enlarged plasmon spectrum from SLG. c) Histogram comparing the plasmon peak position and *σ* ^*^/(*σ* ^*^+*π* ^*^) fraction of VG, VGN, VGNO, and SLG. d) Nitrogen K‐edge EELS spectrum and e) oxygen K‐edge EELS spectrum of VGNO. f) Zero‐loss Energy‐filtered TEM images and EELS elemental mapping images from VGN and VGNO.

Further evidence for local chemical state of C, N, O were obtained using energy‐filtered TEM (EFTEM) and electron energy loss spectroscopy (EELS). **Figure** **3**a shows the EELS spectra at low energy of VG, VGN, VGNO, and perfect single layer graphene (SLG) from high temperature chemical vapor deposition, all being taken under the same acquisition condition. One can see that plasmon peaks are located in a broad region of 15–25 eV for different samples. The peak positions of VG, VGN, and VGNO are shifted gradually to lower energies, from 24.6 to 17.6 eV to approach the peak position of SLG (15.5 eV) (Figure 3b). This is in agreement with the discovery that the plasmon peak is very sensitive to the number of layers and electronic structure,^[^ ^42^ ^]^ and our HREM outcome that the average number of layers in the well‐aligned vertical graphene owing to heteroatom doping. Different doping also strongly affects the C K‐edge characteristics of graphene samples, which represents the transition rate of the valence electron from the 1s core level to the unoccupied antibonding states.^[^ ^43^ ^]^ The sharp peaks located at 285.2 eV is attributed to the adsorption to excite the C 1s core level to *π* ^*^ orbitals typical for sp^2^ bonding, which is absent for diamond C with only the sp^3^ *σ*/*σ* ^*^ orbitals. The broad peak around 292 eV is assigned to absorption by the *σ* ^*^ antibonding orbitals (Figure 3b), which applies to both sp^2^ and sp^3^ C‐C bonding but with difference in fine details. Consequently, enhanced *σ* ^*^ over (*σ* ^*^+ *π* ^*^) is indicative of the presence of the sp^3^ bonding in carbon materials. As is evident in Figure 3c, the fraction of *σ* ^*^ is 61.6% for SLG, and it is increased to 65.2% for VG, 67.5% for VGN, and finally 69.5% for VGNO. For the ideal SLG lattice, the carbon atoms are all in sp^2^ bonding configuration, while in the PECVD samples doping and point defects lead to increased sp^3^ bonding and hence reduced *π* ^*^ but enhanced *σ* ^*^ fractions.^[^ ^36^ ^]^ As is shown in the C K‐edge spectra, nitrogen and oxygen doping of VGNO does result in enhanced *σ* ^*^ fraction. The K‐edge of N and O from VGNO are shown in Figure 3d,e, wherein the *π* ^*^ state corresponds to pyridinic C═N and C═O, respectively, while the *σ* ^*^ state represents the general transition from the core shell to antibonding sp orbitals.^[^ ^44^, ^45^ ^]^ Thereby, the well‐defined *π* ^*^ peaks in the EELS spectra indicate abundant pyridinic N and C═O doping configurations for nitrogen doping and oxygen doping respectively, being consistent with the XPS results. The doping of nitrogen and oxygen tends to alter the local coordination of carbon atoms in graphene lattices due to their larger numbers of valence electrons than that of carbon, thus leading to doped configurations associated with C vacancies and hence sp^3^ orbital components during the advancement of the front graphene edges. Figure 3f shows the zero‐loss EFTEM images and the corresponding EELS elemental mapping images of VGN and VGNO. This further manifests the homogeneous distribution of N and N, O element over the VGN and VGNO for single doping and co‐doping in HPECVD system. Apparently, such uniformly distributed heteroatoms associated with abundant doped configurations are critical to the redox kinetics related to electrochemical processes such as the OER and ORR essential to the performance of metal air batteries and fuel cells.

**Figure 4:**
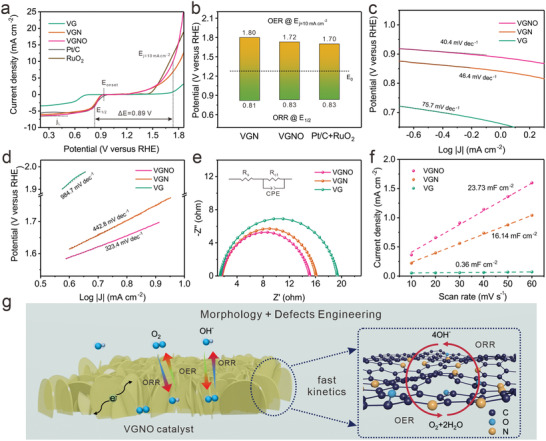
a) LSV polarization curves of different catalysts for ORR and OER in 0.1 m KOH solutions with *iR*‐correction. b) Comparison of potential gap between E_OER_ and E_ORR_ for VGN, VGNO, and Pt/C + RuO_2_. Tafel plots and corresponding slopes of various catalysts for c) ORR and d) OER. e) Nyquist plots of various catalysts (the inset is the equivalent circuit). f) Current density differences at 1.07 V plotted against scan rates and corresponding electrical double‐layer capacitance of various catalysts. g) Schematic representation of synergistic morphology and defects benefits for ORR and OER of VGNO catalysts.

The N and O co‐doping effect on the electrochemical activity for redox processes has been evaluated, in terms of the ORR and OER activity of VG, VGN, and VGNO, using a three‐electrode system with 0.1 m KOH oxygen saturated solutions as electrolyte. The results are compared to the performance of the commercial catalysts based on Pt/C (20 wt%) and RuO_2_ as benchmark references. As is shown in the polarization curves over ORR and OER in **Figure** **4**a, the VGNO catalyst exhibits remarkable ORR activity with an onset potential (*E*_onset_) of 0.92 V, half‐wave potential (E_1/2_) of 0.83 V and a diffusion limiting current density (j_L_) of 6.3 mA cm^−2^ (at 0.3 V), which is comparable to that of commercial Pt/C (E_onset_ of 0.98 V and E_1/2_ of 0.83 V). The ORR electron transfer pathway of VGNO was assessed in the rotating ring‐disk electrode (RRDE) measurements (Figure S8, Supporting Information). Comparing to the Pt/C, VGNO shows a larger disk current for oxygen reduction and a lower ring current for peroxide oxidation (Figure S8a, Supporting Information). Besides, the electron transfer number of VGNO keeps beyond 3.9 with the H_2_O_2_ yield below 5%, which is superior to that of composite catalysts based on CNT^[^ ^46^ ^]^ and close to the performance of the benchmark Pt/C catalyst (Figure S8b, Supporting Information). Meanwhile, the OER performance of VGNO surpasses that of VG and VGN with a low overpotential below 500 mV acquired at 10 mA cm^−2^, which is very close to that for the benchmark RuO_2_ catalyst.

The bifunctional ORR‐OER activity of catalysts was assessed by the potential gap between the OER potential acquired at 10 mA cm^–2^ and half‐wave potential of ORR (Figure 4b). Generally, a smaller potential gap indicates superior oxygen catalytic activity.^[^ ^47^ ^]^ As shown in Figure 4b, VGNO yields a low potential gap value of 0.89 V, outperforming single doped VGN and being comparable to the mixed catalyst Pt/C + RuO_2_. VGNO also gives a smaller Tafel slope with respect to VGN and non‐doped samples, indicating accelerated electrochemical reaction kinetics of ORR and OER (Figure 4c, d).^[^ ^48^ ^]^ The superior kinetics was also verified by the electrochemical impedance spectroscopy (EIS), with the smallest electron transfer resistance (*R*_ct_) during the ORR process (Figure 4e). Electrochemical surface areas (ECSA) of VG, VGN, and VGNO were estimated by the double layer capacitance (C_DL_) calculated via CV measurement at different scan rates (Figure S9, Supporting Information). The C_DL_ of VGNO is as large as 23.73 mF cm^−2^, compared with the low value of 16.14 mF cm^−2^ for VGN and 0.36 mF cm^−2^ for VG, as shown in Figure 4f. This indicates that the ECSA of VGNO is higher than VG and VGN. The higher ECSA could also indicate porous and well‐exposed graphene surficial structure,^[^ ^49^ ^]^ which is indicative of the population of electrochemically active sites.^[^ ^50^ ^]^ Overall, such data are demonstrative of the advantageous morphologies and preferable doping configurations of the co‐doped VGNO, which help enable fast redox kinetics behind the outstanding ORR and OER performances without using precious and expensive Pt and Ru resources (Figure 4g).

**Figure 5:**
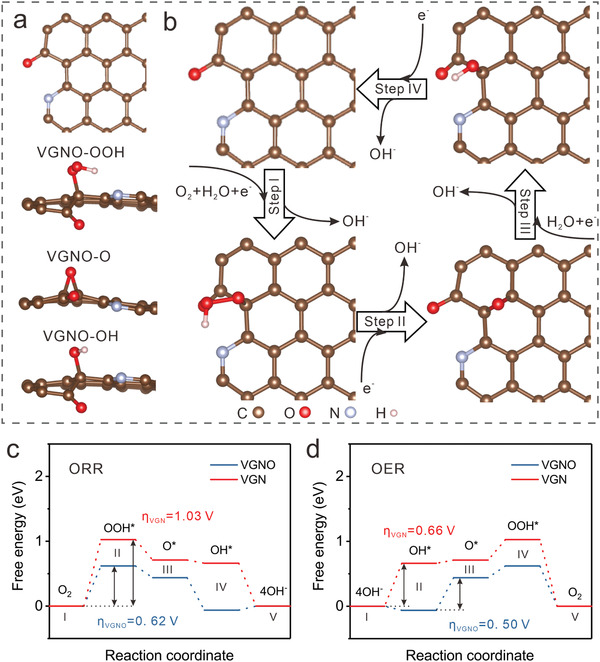
a) The optimized structures before and after the adsorption of OOH*, O*, and OH* intermediates on VGNO. b) ORR pathway occurred on optimized VGNO structure in alkaline solutions. Energy profiles for c) ORR and d) OER pathway of VGNO in the alkaline media (pH = 13) at the equilibrium potential (U = 0.46 V).

The bi‐functional activity of VGNO catalysts has been investigated using the density functional theory (DFT) method. **Figure** **5**a shows the energetically preferred structures before and after the adsorption of OOH^*^, O^*^, and OH^*^ intermediates on VGNO, from DFT energy minimization. The low energy configurations for nitrogen and oxygen dopants are compared (Figure S10, Supporting Information), showing that the minimal energy states for both are pyridinic with hence least out‐plane distortion in the graphene bonding, being consistent with our XPS and EELS analyses. The minimal energy configurations after the adsorption of related intermediates for VGN are shown in Figure S11 (Supporting Information). The ORR and OER pathways in alkaline solutions occurred on VGNO is demonstrated in Figure 5b and Figure S12 (Supporting Information), respectively. The energetic profiles involved for ORR and OER processes in alkaline electrolyte are compared for VGN and VGNO in Figure 5c,d and Figure S13 (Supporting Information). For ORR, the first step OOH^*^ formation is uphill (endothermic process) while other steps are downhill (exothermic process) when the applied potential is U = 0 V (Figure S13, Supporting Information). The energy barrier will be effectively reduced under a positive applied potential, and one notes that with the consideration of a moderate U of 0.46 V, the barrier for VGNO is remarkably smaller than that of the VGN for ORR (0.62 vs 1.03 V), which is fundamental to a significantly faster ORR kinetics of VGNO than of VGN. On the other hand, the OER process is uphill for the first three steps, with the first OH^−^ to OH^*^ step being the controlling or potentially determining step (PDS) for VGNO and the second OH^*^ to O^*^for VGN. At the equilibrium potential (Figure 5c,d), one can see that both the PDS and overall potential barriers for the OER are smaller in the VGNO than in the VGN. For the PDS step, the barriers for VGNO and VGN are 0.50 versus 0.66 eV, while the differences in the overall barriers for the OER process in VGNO and VGN are much bigger, being 0.68 and 1.03 eV, respectively. Fundamentally, the overpotentials for both ORR and OER can be significantly reduced and even eliminated with increasing U, and the results at a U being only 0.46 V can therefore be considered as minimal theoretical overpotentials,^[^ ^51^, ^52^ ^]^ with the above modelling results being consistent with significantly enhanced ORR and OER kinetics in alkaline electrolytes media. Thus, lower overpotential indicates superior electrochemical performance.

**Figure 6:**
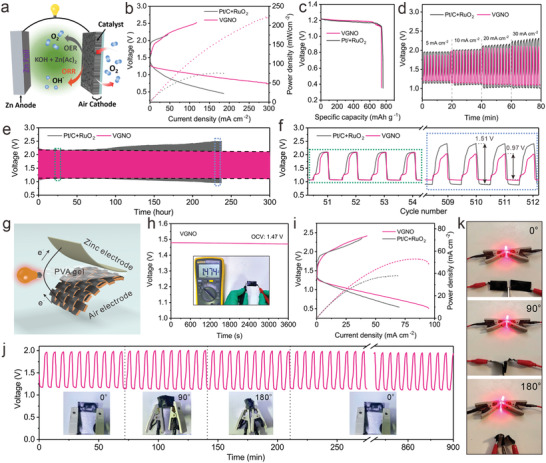
a) Schematic illustration of an aqueous rechargeable ZAB (A‐ZAB) using VGNO coated fiber paper as the air cathode. b) Discharge/charge polarization (solid lines) and corresponding power density (dotted lines) curves, c) galvanostatic discharge curves, d) rate performance tested at 10 mA cm^−2^ of VGNO and Pt/C + RuO_2_ based rechargeable A‐ZABs. e) Galvanostatic discharge/charge cycling curves at 10 mA cm^−2^ of VGNO and Pt/C + RuO_2_ based A‐ZABs. f) Enlarged Galvanostatic charge–discharge cycling curves in e). g) Schematic representation of the solid‐state and flexible ZAB (S‐ZAB). h) Open‐circuit voltage measurement of the S‐ZAB with VGNO catalyst. i) Discharge/charge polarization and corresponding power density curves of VGNO and Pt/C + RuO_2_ based rechargeable S‐ZAB. j) Galvanostatic discharge/charge cycling performance of the S‐ZAB with VGNO catalyst at different bending states. k) Photos of a lighted LED powered by two S‐ZABs in series under different bending states.

In view of its excellent ORR/OER, the VGNO has been applied to aqueous Zn–air battery (ZAB) as catalyzed cathode. The assembled rechargeable aqueous ZAB (A‐ZAB) is illustrated in **Figure** **6**a. For catalyzed cathode, VGNO was spray coated onto conductive paper of carbon fibers, which penetrated tens of micrometers (Figure S14, Supporting Information). The hydrophilicity of hydroxides introduced by oxygenizing plasma enhances the alkali electrolyte infiltration into the VGNO loaded carbon paper. The porous carbon paper allows penetration of O_2_ into the catalyzed cathode wherein the in‐depth loaded graphene provides numerous surficial sites for redox catalysis. As was demonstrated recently, in‐depth loading of catalysts in the air cathode is an effective means in establishing a well‐connected multiphase system across the electrolyte‐catalyst‐carbon paper interfaces, which is beneficial for continuous transportation of hydroxyl anions.^[^ ^53^ ^]^

Curves for discharge/charge polarization (solid lines) and corresponding power density (dotted lines) are shown in Figure 6b. The VGNO based A‐ZAB displays an evidently smaller discharge/charge voltage gap. At 1 V, VGNO battery exhibits a significantly larger discharge current density almost four times of that for the battery using Pt/C + RuO_2_, 120.9 mA cm^−2^ versus 32.3 mA cm^−2^, with a constant high open circuit voltage of 1.48 V being maintained (Figure S15, Supporting Information). Meanwhile, the VGNO based A‐ZAB exhibits a high peak areal power density of 221.9 mW cm^−2^, which is much higher than 75.9 mW cm^−2^ for Pt/C + RuO_2_. Full‐discharge curves at 10 mA cm^−2^ was also plotted and shown in Figure 6c. At a discharge current of 10 mA cm^−2^, the specific capacity (against the mass of consumed zinc) and the energy density of the VGNO based A‐ZAB reaches up to 740.2 mAh g^−1^ and 876.7 Wh kg^−1^ respectively, which rivals the performance of the benchmark Pt/C + RuO_2_ based battery cell (760.8 mAh g^−1^ and 898.4 Wh kg^−1^ for Pt/C + RuO_2_), Figure 6c; and S16 (Supporting Information). The nearly rectangular galvanostatic discharging curve closely matches that of the benchmark battery cell based on expensive catalysts using rare metals is highly attractive for batteries, since their potentially huge demand makes sustainability of resources a necessary concern.

For rate performance in terms of the voltage gap between charge and discharge, Figure 6d, the VGNO A‐ZAB gets more and more advantageous than the benchmark Pt/C + RuO_2_ counter device over increasing current density. While the voltage gap enlarges with increasing current density due to polarization, the graphene‐based system outperforms the Pt/C + RuO_2_ cell, thanks to the faster redox kinetics of the former cell.

Long‐term galvanostatic discharge/charge performance at 10 mA cm^−2^ (10 min for discharge and 10 min for charge) is presented in Figure 6e, and one can see that the VGNO A‐ZAB demonstrates very good stability in holding the charge‐discharge voltage gap over prolonged discharge–charge cycles. An almost constant discharge/charge voltage gap of 0.97 V (1.05 V of discharge and 2.02 V of charge) was maintained over 300 h, while in huge contrast, the Pt/C + RuO_2_ based A‐ZAB experienced significant polarization up to 230 h cycling with the voltage gap increased from 1.04 to 1.51 V when it stopped functioning. Overall, comparing to the benchmark Pt/C + RuO_2_ battery cell, the metal‐free VGNO cell enabled big open circuit voltage, superior discharge/charge stability, and higher discharge power density. Such a metal‐free VGNO based Zn–air system is also superior to most recently reported A‐ZABs including both carbon‐based^[^ ^54^, ^55^ ^]^ and metal‐based cathode^[^ ^48^, ^56^ ^]^ (Table S1, Supplementary Information). The current A‐ZAB based on VGNO dual‐functional catalyst has a highest open circuit voltage of 1.48 V and delivers a highest areal power density (222, vs 93–212 mW cm^−2^) at the same catalyst loading (1 mg cm^−2^),^[^ ^13^, ^48^, ^53^, ^54^, ^56^ ^]^ which is very close to the other champion cells (185–260 mW cm^–2^) with twice loading of catalysts (2 mg cm^−2^).^[^ ^55^, ^57^, ^58^ ^]^ It is worth pointing out that an initial open circuit voltage of 1.48 V is very close to the DFT predicted limit against the Zn/Zn^+2^ anode, which is associated with the electrochemical potential from turning one mole of Zn into ZnO with reference to crystalline Zn and the oxygen gas (1.45 V, without considering the entropy effect). Understandably, the effective voltage over prolonged cycling could be somewhat lowered, since the spontaneous tendency in covering the Zn surface by zinc hydroxide will decrease the theoretical open circuit voltage down to 1.27 V.

The co‐doping with oxygen in the VGNO also promotes reversable redox at the cathode. The VGNO catalyst on the cathode was examined by XPS analysis, before and after long‐term cycling (Figure S17, Supporting Information). It is apparent that the C 1s spectrum does not show evident change, after the prolonged charge‐discharge cycling. On the other hand, cycling does have considerable effect on the N 1s spectrum. It results in decreased content of pyridinic N from 42.4% to 31.7% and appearance of a new peak located at a high binding energy of 406.1 eV, which is attributed to the transition of pyridinic N to oxidized state. This explains the decreased discharge and charge voltage after long‐term cycling. This is in accord with previous reports that an applied positive potential at the cathode in the charging process tends to oxidize the pyridinic N and thus compromise the effect on doped nitrogen on the ORR activity.^[^ ^58^, ^59^ ^]^ The observed resilience in the voltage gap over continuous charging and discharging suggests that such a selective oxidizing does not impact on the VGNO as much as in solely N‐doped carbon materials, since the redox process is largely associated with the pyridinic oxygen (Figure 5a; and Figure S11, Supporting Information). Consequently, outstanding cycling stability is maintainable in the current VGNO based Zn‐air batteries.

To demonstrate the application in wearable/flexible applications, we have assembled solid‐state ZABs (S‐ZABs) with VGNO sprayed coated carbon fiber paper as the air cathode, polyvinyl alcohol (PVA) hydrogel as the electrolyte, and polished zinc foil as anode, as shown in Figure 6g. The as‐assembled S‐ZAB exhibits a stable open‐circuit voltage of 1.47 V in the flat state (Figure 6h), which is higher than most recently reported S‐ZABs (Table S2, Supplementary Information). Figure 6i shows the discharge‐charge polarization and corresponding power density curves of VGNO and Pt/C + RuO_2_ based S‐ZAB. The VGNO exhibits a low voltage gap between discharge and charge process, with a peak power density of 52.9 mW cm^−2^, which is superior to that of the Pt/C + RuO_2_ (38.0 mW cm^−2^). To evaluate the flexibility of the VGNO based S‐ZAB, we conducted galvanostatic discharge‐charge cycling test at different bending angles of 0°, 90°, and 180° at a current density of 3 mA cm^−2^ (Figure 6j). Notably, no obvious discharge/charge deterioration was observed during the bending process. After recovering to flat stage, the battery even exhibited a stable cycling performance with a discharge‐charge voltage gap of 0.83 V (1.12 V of discharge and 1.95 V of charge) for over 900 min. Furthermore, we successfully powered a LED through two VGNO based S‐ZABs in series under different bending stages (Figure 6k). Similarly, no obvious brightness difference was observed when bending at 0°, 90°, and 180°, further confirming the excellent flexibility of VGNO based S‐ZAB.

## 3 Conclusion

We have established an in situ plasma diagnostics setup within our recently developed HPECVD process that utilizes streamlined magnetic field to enhance and maintain uniform high‐density plasma over extended space. This enables real‐time monitoring of the depositing species, so that effective codoping of N and O is realized in densely populated vertical few‐layer graphene sheets (VGNO), through process optimization for beneficial plasma constituents.

The high‐flux plasma containing uniformly excited radicals enables high reactivity in forming the sp^2^ type C—C bonding, which promotes formation of codoping configurations in line with theoretically predicted minimal energy states. Being consistent with DFT modeling, such doped states are mainly at pyridinic sites next to C vacancies in the graphene structure. Their high population in the few‐layer graphene is very active in promoting the ORR‐OER redox process, which is key to successful application as VGNO catalyzed Zn–air batteries.

The current work provides a highly attractive route for developing high‐performance rechargeable Zn‐air batteries using carbon‐only air cathode, which are more stable than battery cells with benchmarked industrial catalysts based on rare metal resources such as Ru and Pt. In addition to superb power density, large open circuit voltage, minimal polarization over charging‐charging, and high cycling stability, the current system can be readily made into flexible solid batteries.

## Conflict of Interest

The authors declare no conflict of interest.

## Supporting information

Supporting Information

## Data Availability

The data that support the findings of this study are available from the corresponding author upon reasonable request.
