# Supplementary material for: In Situ Monitored (N, O)‐Doping of Flexible Vertical Graphene Films with High‐Flux Plasma Enhanced Chemical Vapor Deposition for Remarkable Metal‐Free Redox Catalysis Essential to Alkaline Zinc–Air Batteries
Source: Adv Sci (Weinh). 2022 Mar 4;9(13):2200614. doi: 10.1002/advs.202200614 (PMC9069200; doi:10.1002/advs.202200614)
Supplement: Supplementary file 1 — Supporting Information [file ADVS-9-2200614-s001.pdf]

Supporting Information

In Situ Monitored (N, O)-doping of Flexible Vertical Graphene Films with High-Flux Plasma Enhanced Chemical Vapor Deposition for Remarkable Metal-free Redox Catalysis Essential to Alkaline Zinc–Air Batteries

Zhiheng Wu, Yuran Yu, Gongkai Zhang, Yongshang Zhang, Ruxin Guo, Lu Li, Yige Zhao, Zhuo Wang, Yonglong Shen, Guosheng Shao**

Zhiheng Wu, Yuran Yu, Gongkai Zhang, Yongshang Zhang, Ruxin Guo, Lu Li, Dr. Yige Zhao, Dr. Zhuo Wang, Dr. Yonglong Shen, Prof. Guosheng Shao
State Center for International Cooperation on Designer Low-carbon & Environmental Materials (CDLCEM), School of Materials Science and Engineering, 100 Kexue Avenue, Zhengzhou University, Zhengzhou 450001, China
E-mail: gsshao@zzu.edu.cn; shenyonglong@zzu.edu.cn
*Z Wu and Y Yu contribute equivalently to this work

Zhiheng Wu, Yuran Yu, Yongshang Zhang, Ruxin Guo, Dr. Yonglong Shen, Prof. Guosheng Shao
Zhengzhou Materials Genome Institute (ZMGI), Building 2, Zhongyuanzhigu, Xingyang, Zhengzhou 450100, China

Methods

Experimental

Growth of vertical graphene films: Vertical graphene films were fabricated in our previously reported high-flux plasma enhanced chemical vapor deposition (HPECVD) system. All substrates were firstly thoroughly cleaned ultrasonically in acetone, ethanol, and deionized water. Subsequently, the substrates were placed on the holder. After the pressure of the vacuum chamber was pumped below 5×10^{-4} mbar, the Ar gas (50 sccm) was fed into the chamber and the plasma was launched to etch off the surface contaminant for 5 min. Then, CH_4, N_2, and O_2 were introduced into the chamber without shutting off the plasma. Undoped vertical graphene films (VG) and doped vertical graphene films (VGX) can be easily obtained by varying the gas flow rates of the introduced gas.

In situ plasma diagnostics system: The *in situ* plasma diagnostic system was customized with a Langmuir probe (ALP 150, Impedans) and a multichannel spectrometer (AvaSpec-ULS3648, AVANTES). The Langmuir probe was equipped with a single-probe configuration and conducted in a multi-scan mode to monitor the electron density of the plasma. Meanwhile, the optical emission from the plasma was transmitted via an optical fiber to the multichannel spectrometer to monitor the radicals in the plasma produced during growth.

Finite element modeling and simulation: Simplified 2D models of HPECVD system based on COMSOL Multiphysics software were constructed to simulate both the electrical field and electron density distribution. The scale of each component in models was proportional to the real equipment. The RF coil power was set to 1700 W. The current of electromagnetic coil 2 (Ec2) was set to be twice larger than that of Ec1. The initial electron density of plasma was set to 1×10^{15} 1/m^3. The temperature and absolute pressure of the plasma model were set to 300 K and 0.7 Pa.

Materials characterization: The morphologies and detailed structures of samples were investigated by SEM (ZEISS SIGMA 500, operating at 5 kV attached with a Bruker energy dispersive X-ray spectrometer). TEM and STEM were operated at 200 kV with a cold field emission source (FEI Tecnai G2 F20), Raman (Horiba LabRAM HR Evolution Spectrometer, with a 532 nm excitation wavelength), XPS (AXIS Supra, all data were calibrated with the C 1s of 284.6 eV). Electron energy loss spectra (EELS) data were collected to clarify the

chemical state and structure of VGNO. The EELS experiment was conducted in the diffraction mode using the Tecnai G2 F20 TEM equipped with an imaging filter (GIF Quantum 965, Gatan, Inc) operated at 200kV. The camera length was 100 mm and the entrance aperture was 5 mm. EELS were acquired at an energy dispersion of 0.1 eV/channel using a collection half-angle of 17.4 mrad. The energy resolution at the zero-loss peak was approximately 0.9 eV. The low-loss and core-loss regions were collected separately for all specimen regions studied. The specimens were tested from the selected regions with relative thickness of $t/\lambda < 0.4$. Energy filtered TEM was operated under the EELS conditions. Zero-loss images of specimen regions studied were obtained with a 10 eV energy slit. The C K-edge, N K-edge and O K-edge were used to form the EELS-images to study the light elements distribution in the specimens area selected.

Electrochemical measurements: Electrochemical tests were performed using a CHI760E electrochemical workstation (Shanghai, China) in a normal three-electrode configuration. A pre-polished rotating disk glassy carbon electrode (RDE) and rotating ring-disk glassy carbon electrode (RRDE) loaded with catalyst was used as the working electrode. A KCl saturated Hg/Hg_2Cl_2 electrode (SCE) and a graphite rod were used as the reference electrode and counter electrode, respectively. The potential was calculated to a reversible hydrogen electrode (RHE): $E_{RHE} = E_{SCE} + 0.241 \text{ V} + 0.059 \times \text{pH}$. Oxygen saturated 0.1 M KOH was used as the electrolyte for ORR and OER test.

For ORR and OER tests, the working electrode was prepared by firstly dispersing catalyst (5 mg) in ethanol (950 ul) and 5 % Nafion solution (50 ul) via ultrasonication. Then, the catalyst ink was transferred on glassy carbon electrode. The mass loading of catalyst was controlled to 0.47 mg cm^{-2}. For comparison, Pt/C (20 wt%) and RuO_2 catalysts were also prepared in the same procedure. Cyclic voltammetry (CV) measurement was conducted at a scan rate of 50 mV s^{-1} and the liner sweep voltammetry (LSV) was recorded at a scan rate of 5 mV s^{-1} with 95 % *iR*-compensation.

The electron transfer number and the percentage of HO_2^- in oxygen reduction process for ORR were calculated using equations:

$$n = 4 \times \frac{I_D}{\frac{I_R}{N} + I_D} \quad (1)$$

$$HO_2^-\% = \frac{200 \times I_R}{N \times I_D + I_R} \quad (2)$$

where I_D represents the disk current and I_R represents the ring currents, the ring collection efficiency (N) is calculated to be 0.424.

The electrochemical surface area (ECSA) was measured in the non-Faradaic current during cycling the electrode. The current density scale linearly with the scan rate (v), thus the double-layer capacitance (C_{dl}) and ECSA can be determined by equations:

$$C_{dl} = \frac{j}{v} \quad (3)$$

$$ECSA = \frac{C_{dl}}{C_{ref}} \quad (4)$$

where j is the current density recorded in the non-Faradaic region, C_{ref} is determined to be 0.047 mF/cm^2 of the glassy carbon electrode.

Battery assembling: Aqueous rechargeable zinc–air batteries (A-ZABs) were assembled using plastic electrochemical cells with a polished Zn anode and an air cathode in the mixture electrolyte of 6 M KOH and 0.2 M $Zn\ (Ac)_2$. The cathode was composed of a gas diffusion layer, a current collector layer, and a spray coated catalyst layer. A mixture of 20 wt% Pt/C and RuO_2 with a mass ratio of 1:1 was also prepared in catalyst layer as reference. The active area is 1 cm^{-2} and the mass loading is controlled at 1.0 mg cm^{-2}. All the measurements were conducted in a CHI760E electrochemical workstation and a Land CT2001A battery testing station at ambient environment.

Our flexible solid-state ZABs (S-ZABs) were integrated in a planar configuration of a hydrogel electrolyte membrane sandwiched between a zinc foil (80 um in thickness) and a catalyst spray coated flexible carbon cloth. The active area is 1 cm^{-2} and the mass loading is controlled at 1.0 mg cm^{-2}. All the measurements were conducted in a CHI760E electrochemical workstation and a Land CT2001A battery testing station at ambient environment.

The hydrogel electrolyte membrane was prepared as follows: 1.0 g of polyvinyl acetate powder (PVA, 1799) was dissolved in 10 mL H_2O followed by magnetic stirring for 1 h at 95°C in an oil bath. Then, 1.0 mL of 18 M KOH with 0.1 M $ZnAc_2$ was added into the solution followed by stirring for another 1 h at 95°C in an oil bath. Next, the gel was poured into a homemade container and frozen at -5°C for at least 12 h and the hydrogel was stored at 5°C before use.

Computational simulation

Theoretical calculations were performed in the framework of the density functional theory (DFT), using the Vienna Ab Initio Simulation Package (VASP),[1,2] with the projector augmented wave (PAW) method with a cutoff energy of 500 eV for the ionic potentials

including the overall effect of core electrons.[3,4] For the geometric relaxation of the structures, summation over the Brillouin Zone (BZ) is performed with 2 × 2 × 2 Monkhorst-Pack k-point meshes for the N-O plane (55 atoms). Optimized structures were obtained by minimizing the forces on each ion until they fell below 0.05 eV/Å. All calculations were spin-polarized.

Thermochemistry of the electrochemical oxygen reduction reaction (ORR) and oxygen evolution reaction (OER) was modeled by applying the computational hydrogen electrode method.[5] This method has previously been proved successful in predicting ORR and OER activity trends on various catalysts. Briefly, the Gibbs free energy change of each electrochemical elementary step for the ORR and OER was calculated with DFT. The ORR reaction mechanism was assumed to follow the four-step associative mechanism represented in the following equations:

$$O_2 + H^+ + e^- + {}^* \leftrightarrow OOH^* \qquad \text{I}$$

$$OOH^* + H^+ + e^- \leftrightarrow O^* + H_2O \qquad \text{II}$$

$$O^* + H^+ + e^- \leftrightarrow OH^* \qquad \text{III}$$

$$OH^* + H+ + e^- \leftrightarrow H_2O + {}^* \qquad \text{IV}$$

The overall ORR could be expressed as:

$$O_2 + 4H^+ + 4e^- \rightarrow 2H_2O \qquad (5)$$

By setting the reference potential to be the standard hydrogen electrode, the free energy of $1/2H_2$ can be used to replace that of (H^+ + e^-) with the condition of PH = 0 and U = 0 V (the electrode potential). The free energy of O_2 was obtained from the reaction $2H_2O \rightarrow O_2 + 2H_2$ for which the free energy change of experimental data is 4.92 eV. The standard hydrogen electrode of ORR and OER progress is 1.23 V.

Hence, the Gibbs free energy of each reaction step for ORR can be expressed as：

$$\Delta G_1 = G_{OOH*} - G_{H_2}/2 - eU - G^* - G_{O_2} \qquad (6)$$

$$\Delta G_2 = G_{O*} + G_{H_2O} - G_{H_2}/2 - eU - G_{OOH*} \qquad (7)$$

$$\Delta G_3 = G_{OH*} - G_{H_2}/2 – eU - G_{O*} \qquad (8)$$

$$\Delta G_4 = G_{H_2O} + G^* - G_{H_2}/2 - eU - G_{OH*} \qquad (9)$$

For each adsorption configuration, the Gibbs free energy should be corrected for the adsorption groups will vibrate at different orientations. The corrected value, ΔE is obtained through VASPKIT software by the following equation:

$$\Delta E = ZPE - TS \qquad (10)$$

where ZPE, T and S are the zero-point energy, temperature, and entropy.

With this approach, the theoretical overpotential of ORR (η_{ORR}) at standard conditions is defined as:

$$\eta_{ORR} = 1.23 + \max(\Delta G_1, \Delta G_2, \Delta G_3, \Delta G_4)/e \quad (11)$$

Similarly, the Gibbs free energy of each reaction step for OER can be expressed as:

$$\Delta G_5 = G_{OH*} + G_{H_2}/2 + eU - G^* - G_{H_2O} \quad (12)$$

$$\Delta G_6 = G_{O*} + G_{H_2}/2 + eU - G_{OH*} \quad (13)$$

$$\Delta G_7 = G_{OOH*} + G_{H_2}/2 + eU- G_{O*} - G_{H_2O} \quad (14)$$

$$\Delta G_8 = G_{O_2} + G^* + G_{H_2}/2 + eU - G_{OOH*} \quad (15)$$

The theoretical overpotential of OER (η_{OER}) is defined as:

$$\eta_{OER} = \max(\Delta G_5, \Delta G_6, \Delta G_7, \Delta G_8)/e - 1.23 \quad (16)$$

In addition, the free energy of reaction can be effected by pH when H^+ or OH^- participate in the reaction with a $\Delta G_{pH} = -kT\ln(\frac{1}{|H^+|}) = 0.0592 \times pH$. Taking ORR reaction as an example:

$$O_2 + 4H^+ + 4e^- \rightarrow 2H_2O \quad (17)$$

$$\Delta G = 2G_{H_2O} - [4G_{H^+} + 4G_{e^-} + G_{O_2}] = 2G_{H_2O} - [4(\frac{1}{2}G_{H_2} - 0.0592pH) + G_{O_2}] \quad (18)$$

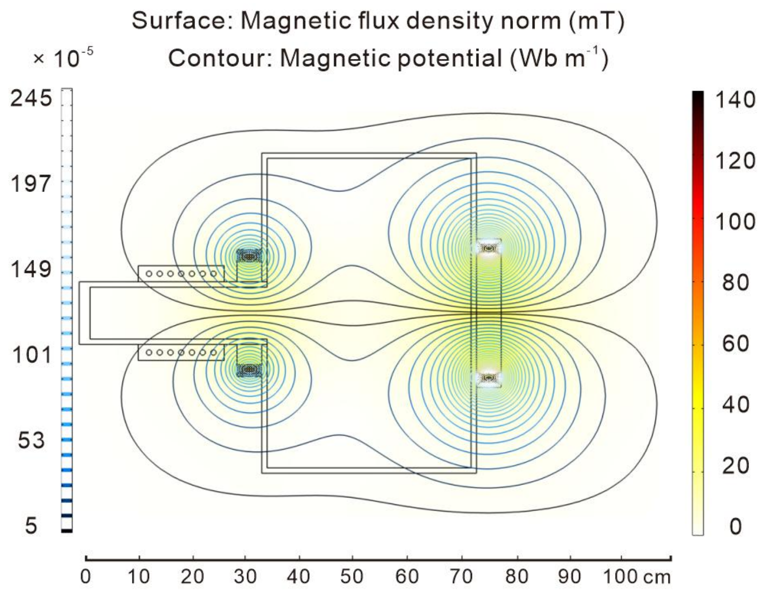

Figure S1. Simulated magnetic field in HPECVD system.

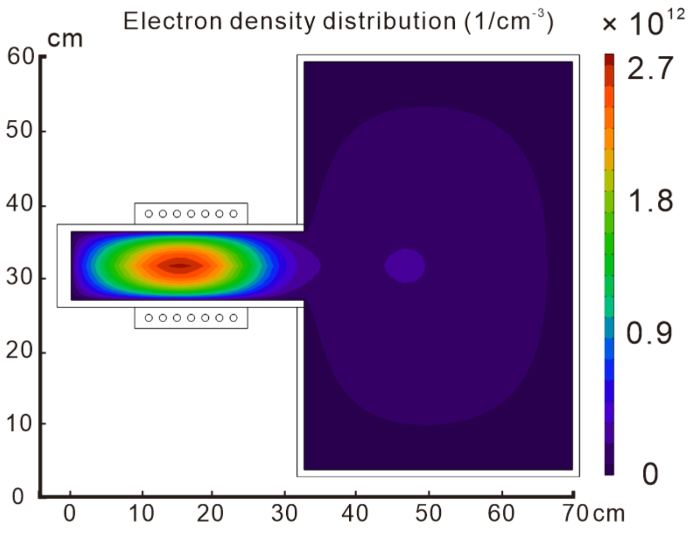

Figure S2. Simulated electron density distribution in a normal PECVD system.

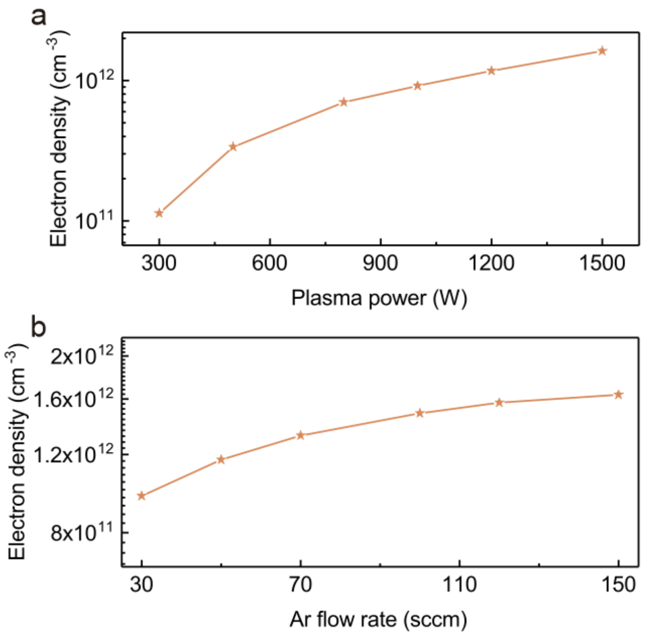

Figure S3. Electron density as a function of a) plasma power and b) Ar flow rate.

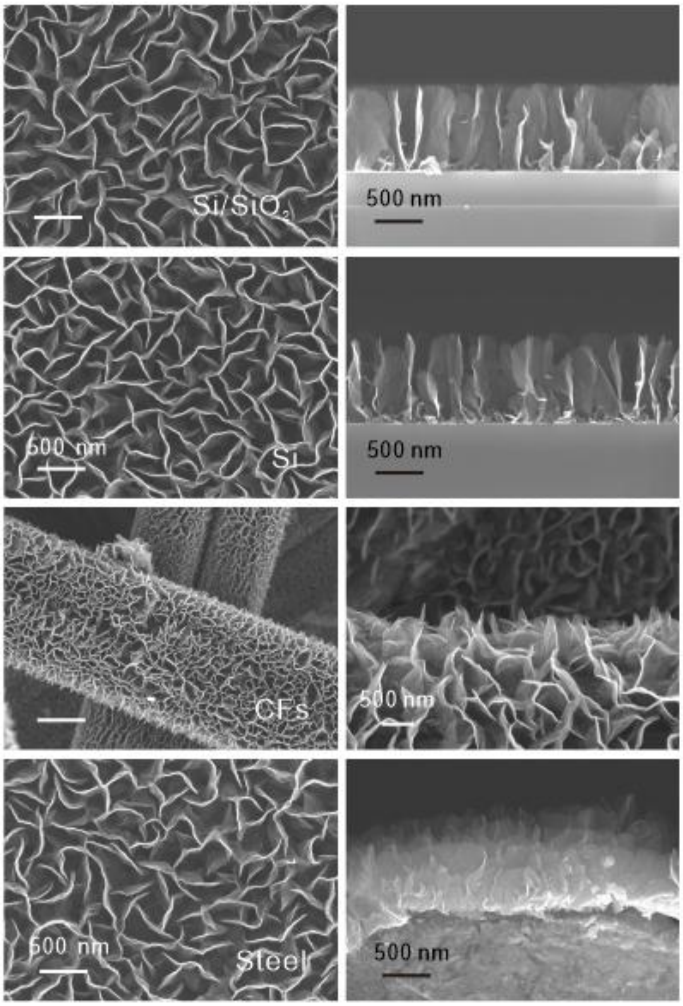

Figure S4. SEM images of VG grown on different substrates in HPECVD system.

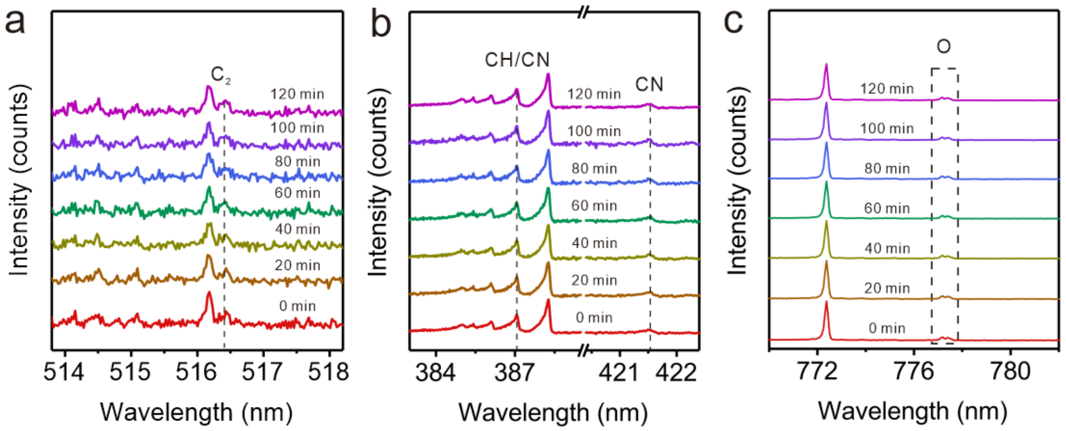

Figure S5. *In situ* OES of Ar/CH_4/N_2/O_2 plasma from 0 to 120 min.

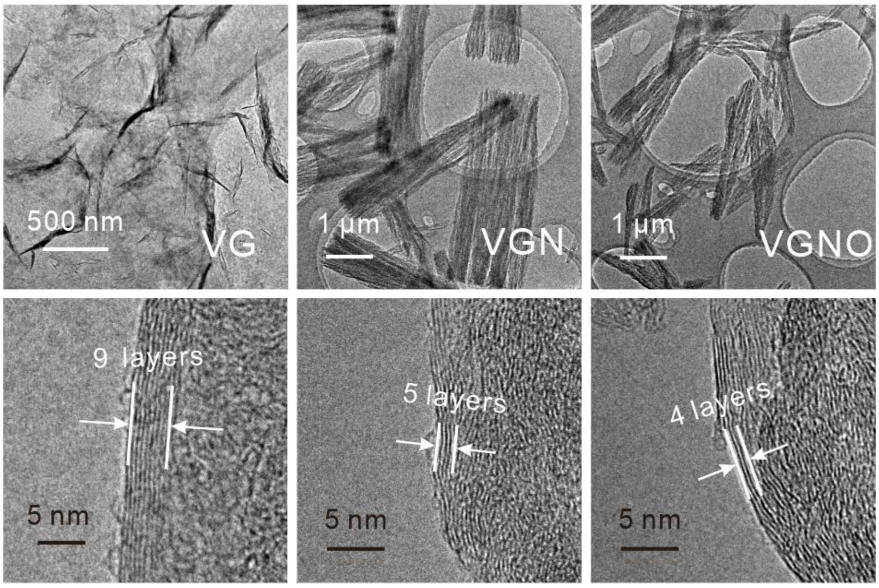

Figure S6. Low-magnification TEM and HRTEM images of VG, VGN, and VGNO.

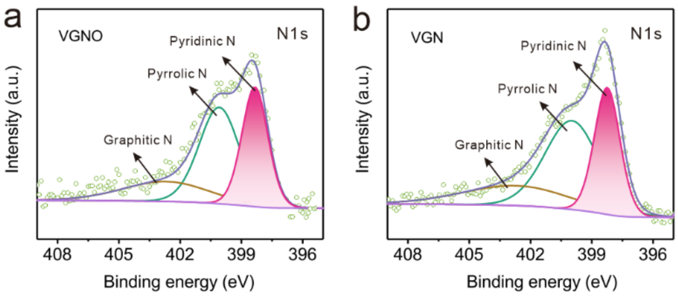

Figure S7. High-resolution XPS spectra of N1s for VGNO and VGN.

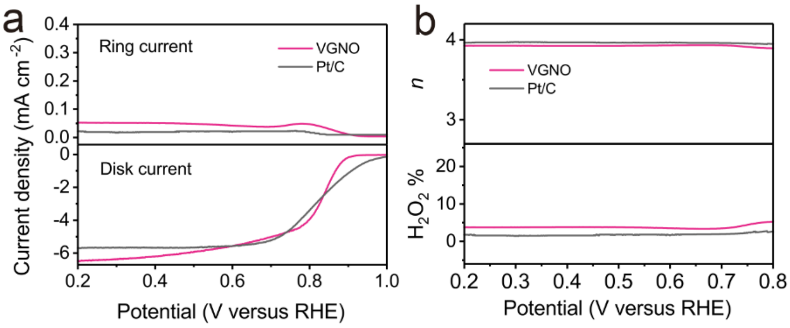

Figure S8. a) Ring currents and disk currents collected in the RRDE system of VGNO and Pt/C catalysts. b) H_2O_2 yield and electron transfer numbers (*n*) of VGNO and Pt/C catalysts.

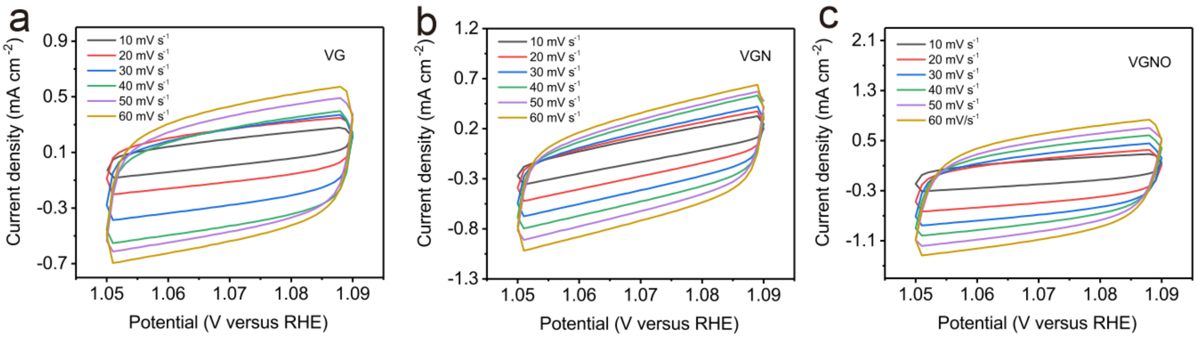

Figure S9. Cyclic voltammograms curves of (a) VG, (b) VGN, and (c) VGNO in the non-Faradaic potential region from 1.05 V to 1.09 V versus RHE with scan rates from 10 to 60 mV s^{-1}.

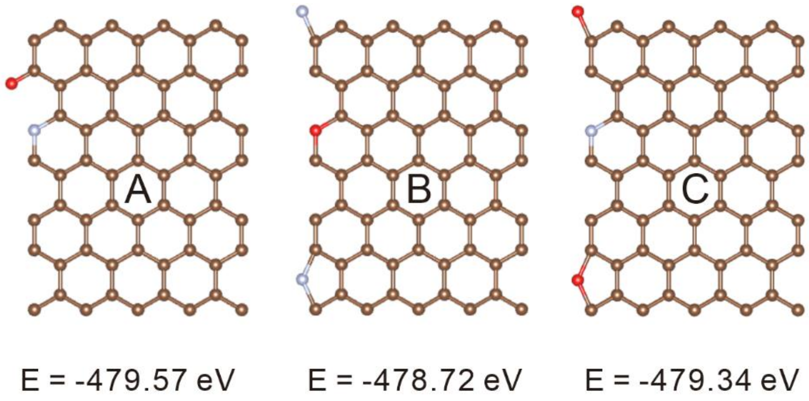

Figure S10. Possible VGNO structures and corresponding total energy obtained from DFT calculation.

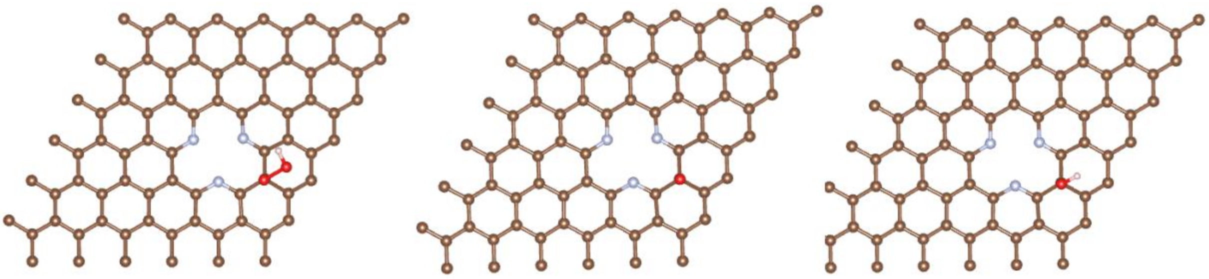

Figure S11. Optimal structure of VGN after the adsorption of oxygen intermediates (OOH*, O* and OH*).

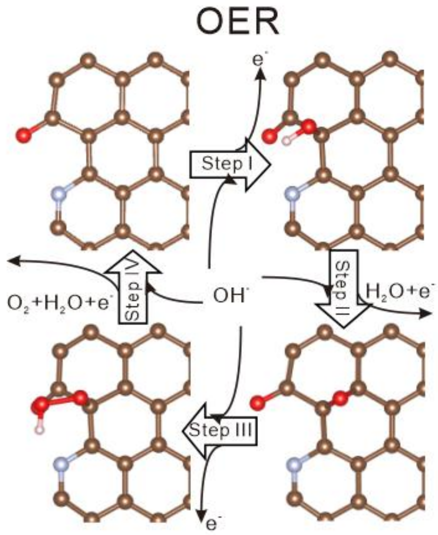

Figure S12. OER pathway occurred on optimized VGNO structure in alkaline solutions.

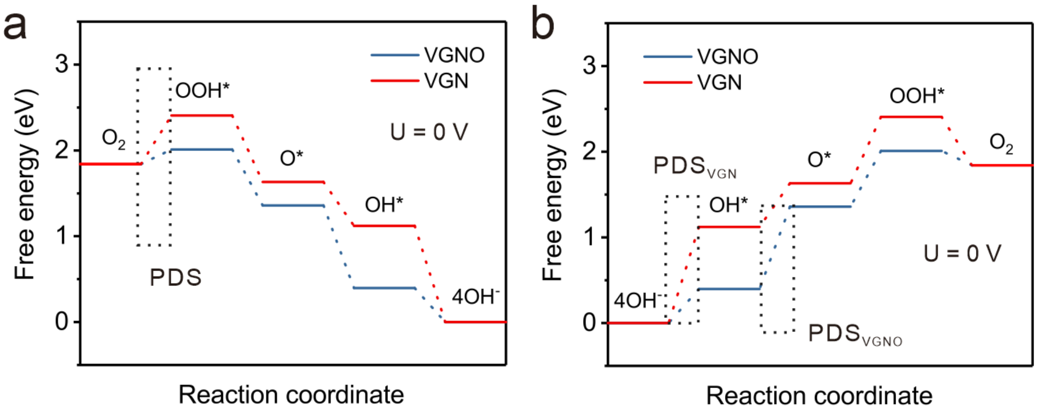

Figure S13. Energy profiles for a) ORR and b) OER pathway of VGNO and VGN in the alkaline media (pH = 13) at the electrode potential of 0 V.

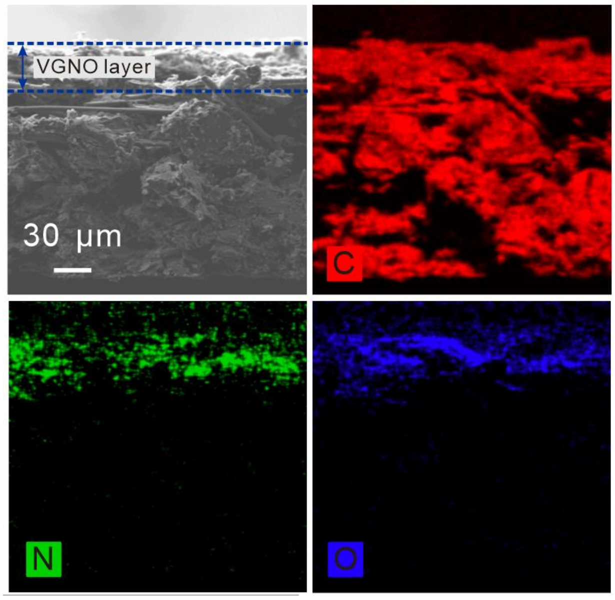

Figure S14. Cross-sectional SEM image of the air cathode of VGNO based ZAB and mapping images of C, N, and O elements.

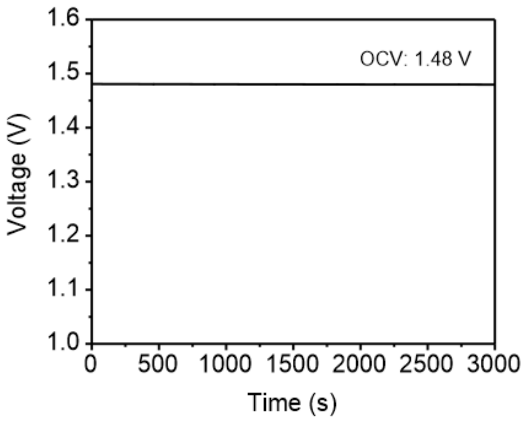

Figure S15. Open-circuit voltage measurement of VGNO based ZAB.

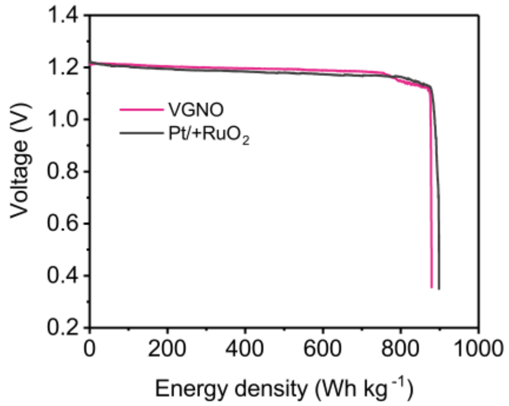

Figure S16. Energy density of ZABs with VGNO and Pt/C + RuO_2 catalysts.

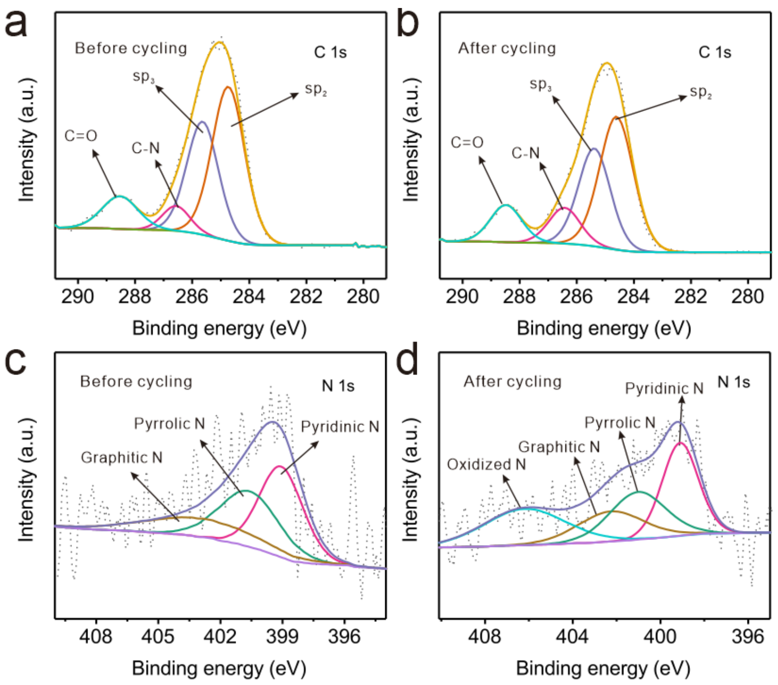

Figure S17. High-resolution XPS spectra of C1s and N1s of VGNO before and after charge/discharge cycling.

Table S1. Performance of recently reported aqueous rechargeable ZABs.

**Catalyst**	**Catalyst Loading (mg cm^{-2})**	**Open circuit voltage (V)**	**Peak power density (mW cm^{-2})**	**Discharge/charge voltage gap @ current density (V) @ (mA cm^{-2})**	**Stability**	**Ref.**
Co_2P@N, P-doped carbon	1.0	1.430	157	0.92 @ 10	140 h @ 10 mA cm^{-2}	*Energy Environ. Mater.* **2021**, DOI: 10.1002/eem2.12208.
N, O-doped graphene like carbon	1.0	1.328	212	0.95 @ 10	370 h @ 10 mA cm^{-2}	*J. Mater. Chem. A* **2020**, *8*, 11202.
NiFe layered-doublehydroxide	1.2	-	93.9	0.86 @ 10	333 h @ 5 mA cm^{-2}	*Adv. Mater.* **2020**, *32*, 1908488.
N-doped carbons	2.0	1.490	192.7	~1.28 @ 10	110 h @ 10 mA cm^{-2}	*Adv. Mater.* **2019**, *31*, 1900341.
FeP/Fe_2O_3@N, P-doped carbon sheets	1.0	-	130	0.89 @ 5	160 h @ 5 mA cm^{-2}	*Adv. Mater.* **2020**, *32*, 2002292.
N-doped carbon nanospheres	1.0	1.48	160	1.1 @ 5	150 h @ 5 mA cm^{-2}	*Energy Storage Mater.* **2020**, *27*, 514.
N, O-doped carbon	2.0	1.45	260.5	0.81 @ 10	388 h @ 10 mA cm^{-2}	*Appl. Catal. B Environ.* **2020**, *265*, 118603.
nanoporous carbon nanofiber films	2.0	1.48	185	0.86 @ 10	83 h @ 10 mA cm^{-2}	*Adv. Mater.* **2016**, *28*, 3000.
N-doped carbon paper with graphene sheet	7	1.43	135	0.98 @ 5	250 cycles @ 5 mA cm^{-2}	*Adv. Energy Mater.* **2018**, *8*, 1703539.
S and N co-doped carbon sheets	-	-	94.8	1.01 @ 10	150 h @ 10 mA cm^{-2}	*J. Mater. Chem. A* **2020**, *8*, 4386.
N, O-doped vertical graphene films	1.0	1.48	221.9	0.97 @ 10	300 h @ 10 mA cm^{-2}	This work

Table S2. Performance of recently reported solid-state rechargeable ZABs.

**Catalyst**	**Electrolyte**	**Open circuit voltage (V)**	**Peak power density (mW cm^{-2})**	**Discharge/charge voltage gap @ current density (V) @ (mA cm^{-2})**	**Stability**	**Ref.**
Oxygen active carbon cloth	PVA with 18 M KOH + 0.1 M $ZnAc_2$	1.367	52.3	0.98 @ 1	1000 min @ 1 mA cm^{-2}	*Adv. Energy Mater.* **2019**, *9*, 1802936.
N, O-doped graphene like carbon	PVA with 9 M KOH	1.328	65.1	~0.48 @ 2	18 h @ 2 mA cm^{-2}	*J. Mater. Chem. A* **2020**, *8*, 11202.
N-doped carbons	cellulose membrane with 6 M KOH + 0.2 M $ZnAc_2$	1.34	36.2	0.77 @ 10	80 h @ 10 mA cm^{-2}	*Adv. Mater.* **2019**, *31*, 1900341.
FeP/Fe_2O_3@N, P-doped carbon sheets	PVA with 18 M KOH + 0.02 M $ZnAc_2$	1.42	40.8	0.98 @ 5	500 min @ 5 mA cm^{-2}	*Adv. Mater.* **2020**, *32*, 2002292.
nanoporous carbon nanofiber films	PVA with 0.1 M KOH + 0.02 M $ZnAc_2$	1.26	-	0.78 @ 2	6 h @ 2 mA cm^{-2}	*Adv. Mater.* **2016**, *28*, 3000.
N-doped carbon paper with graphene sheet	PVA with 0.1 M KOH + 0.02 M $ZnAc_2$	1.335	-	~1.02 @ 1	170 cycles @ 1 mA cm^{-2}	*Adv. Energy Mater.* **2018**, *8*, 1703539.
N-doped carbon layer encapsulated Co and FeCo nanoparticles hybrid nanowire arrays	hydrogel with 11.25 M KOH + 0.25 M ZnO	1.419	82	0.73 @ 1	18 h @ 1 mA cm^{-2}	*Appl. Catal. B Environ.* **2019**, *256*, 117887.
S and N co-doped carbon sheets	PVA with 18 M KOH + 0.1 M $ZnCl_2$	1.39	-	~0.84 @ 1	50 cycles @ 1 mA cm^{-2}	*J. Mater. Chem. A* **2020**, *8*, 4386.
cobalt NPs embedded in N-doped carbon nanotube arrays	PVA with 18 M KOH + 0.02 M $ZnAc_2$	1.484	38.6	0.68 @ 1	19 h @ 1 mA cm^{-2}	*Small Methods* **2020**, *4*, 1900571.
N, S co-doped porous carbon cloth	PVA and PEO with 18 M KOH	1.25	47	1.35 @ 5	480 min @ 5 mA cm^{-2}	*Adv. Sci.* **2018**, *5*, 1800760.
N, O-doped vertical graphene	PVA with 18 M KOH + 0.1 M	1.47	52.9	0.83 @ 2	900 min @	This work

films	$ZnAc_2$		2 mA cm^{-2}

References

[1] G. Kresse, J. Hafner, *Phys. Rev. B* **1993**, *47*, 558.

[2] G. Kresse, J. Hafner, *Phys. Rev. B* **1994**, *49*, 14251.

[3] G. Kresse, D. Joubert, *Phys. Rev. B* **1999**, *59*, 1758.

[4] P. E. Blöchl, *Phys. Rev. B* **1994**, *50*, 17953.

[5] J. K. Nørskov, J. Rossmeisl, A. Logadottir, L. Lindqvist, J. R. Kitchin, T. Bligaard, H. Jónsson, *J. Phys. Chem. B* **2004**, *108*, 17886.
